# Developmental haemostasis in moderate and late preterm infants

**DOI:** 10.1186/1824-7288-40-S2-A38

**Published:** 2014-10-09

**Authors:** Mario Motta, Fabio Giovanni Russo

**Affiliations:** 1 Neonatology and Neonatal Intensive Care Unit, Children’s Hospital of Brescia, Italy

## Introduction

The term “developmental haemostasis” was first coined by Maureen Andrews to describe the age-related physiological changes of the coagulation system during childhood.[1] Given the age-dependent specificity of haemostasis, the evaluation and the interpretation of coagulation assays in newborns may present diagnostic difficulties and appropriate reference ranges for the diagnosis and management of coagulopathies in moderate and late preterm infants are needed.

## Age-related changes in the coagulation plasma proteins

The haemostatic system is a dynamic evolving process that is age-dependent. At birth, plasma concentrations of vitamin K-dependent and contact factors (F) are decreased if compared with adult levels.[2] During the first 6 months of life, they gradually increase to values approaching adult levels.[2] These changes in protein levels lead to corresponding changes in global tests of coagulation such as the Prothrombine Time and the Activated Partial Thromboplastin Time. Plasma concentrations of fibrinogen, FV, FVIII, FXIII and von Willebrand are not decreased at birth.[2] In addition, plasma concentrations of antithrombin, protein C and protein S are low at birth, and they reach adult levels at about 6-12 months of life.[2] In the fibrinolytic system, plasma concentrations of plasminogen are decreased at birth, whereas tissue plasminogen activator and plasminogen activator inhibitor are increased.[2] These postnatal changes in the coagulation system, observed both in term and preterm neonates, are functionally balanced, suggesting a normal haemostasis during early infancy in healthy conditions.

## Reference ranges of coagulation tests in moderate and late preterm infants

Considering the developmental changes of coagulation proteins in term and preterm neonates, specific age-related reference ranges are necessary for an accurate diagnosis and management of neonatal coagulation disorders. In table 1 reference ranges for coagulation assays obtained in moderate and late preterm neonates are summarized.[3] Since coagulation assays are analyzer and reagent dependent, laboratories should develop specific reference ranges to their own testing systems.[4]

**Table 1:** Reference values for coagulation tests in healthy moderate and late preterm neonates (30 to 36 weeks of gestation) during the first 6 months of life.

Postnatal Age	PT (s)	APTT (s)	Fibrinogen (g/L)	AT-III (U/mL)	Protein C (U/mL)	Protein S (U/mL)
Day 1	13.0 (10.6-16.2)	53.6 (27.5-79.4)	2.43 (1.50-3.73)	0.38 (0.14-0.62)	0.28 (0.12-0.44)	0.26 (0.14-0.38)
Day 5	12.5 (10.0-15.3)	50.5 (29.6-74.1)	2.80 (1.60-4.18)	0.56 (0.30-0.82)	0.31 (0.11-0.51)	0.37 (0.13-0.61)
Day 30	11.8 (10.0-13.6)	44.7 (26.9-62.5)	2.54 (1.50-4.14)	0.59 (0.37-0.81)	0.37 (0.15-0.59)	0.56 (0.22-0.90)
Day 90	12.3 (10.0-14.6)	39.5 (28.3-50.7)	2.46 (1.50-3.52)	0.83 (0.45-1.21)	0.45 (0.23-0.67)	0.76 (0.40-1.12)
Day 180	12.5 (10.0-15.0)	37.5 (21.7-53.3)	2.28 (1.50-3.60)	0.90 (0.52-1.28)	0.57 (0.31-0.83)	0.82 (0.44-1.20)

All values are given as a mean followed by lower and upper limit (95% of confidence interval). From Andrew M at al., modified.[3]

## New diagnostic assays

Thromboelastography and the measurement of thrombin generation are methods that provide a global assessment of hemostasis. Recently, the use of these assays has been reported in neonates and the results suggest that these methods may offer advantages for the evaluation of developmental hemostasis. Thromboelastography, in particular, may be less sensitive to age-related changes of coagulation protein.[5,6] However, the introduction of these methods into clinical practice of neonatal medicine should be based on larger studies confirming the predictive value of the assays.
